# Mobile dune fixation by a fast-growing clonal plant: a full life-cycle analysis

**DOI:** 10.1038/srep08935

**Published:** 2015-03-11

**Authors:** Shou-Li Li, Fei-Hai Yu, Marinus J. A. Werger, Ming Dong, Heinjo J. During, Pieter A. Zuidema

**Affiliations:** 1 State Key Laboratory of Vegetation and Environmental Change, Institute of Botany, Chinese Academy of Sciences, Beijing 100093, China; 2 Ecology & Biodiversity Group, Institute of Environmental Biology, Utrecht University, P.O. Box 80084, 3508 TB Utrecht, the Netherlands; 3 College of Nature Conservation, Beijing Forestry University, Beijing 100083, China; 4 Forest Ecology and Forest Management group, Department of Environmental Sciences, Wageningen University, PO Box 47, 6700 AA Wageningen, the Netherlands; 5 Section of Ecology, Department of Biology, University of Turku, 20014 Turku, Finland; 6 College of Life and Environmental Sciences, Hangzhou Normal University, Hangzhou. 310036, China

## Abstract

Desertification is a global environmental problem, and arid dunes with sparse vegetation are especially vulnerable to desertification. One way to combat desertification is to increase vegetation cover by planting plant species that can realize fast population expansion, even in harsh environments. To evaluate the success of planted species and provide guidance for selecting proper species to stabilize active dunes, demographic studies in natural habitats are essential. We studied the life history traits and population dynamics of a dominant clonal shrub *Hedysarum laeve* in Inner-Mongolia, northern China. Vital rates of 19057 ramets were recorded during three annual censuses (2007–2009) and used to parameterize Integral Projection Models to analyse population dynamics. The life history of *H. laeve* was characterized by high ramet turnover and population recruitment entirely depended on clonal propagation. Stochastic population growth rate was 1.32, suggesting that the populations were experiencing rapid expansion. Elasticity analysis revealed that clonal propagation was the key contributor to population growth. The capacity of high clonal propagation and rapid population expansion in mobile dunes makes *H. laeve* a suitable species to combat desertification. Species with similar life-history traits to *H. laeve* are likely to offer good opportunities for stabilizing active dunes in arid inland ecosystems.

Desertification is considered as one of the most critical ecological and environmental issues worldwide, and has gained increasing attention^1^. Inland dunes in arid and semi-arid regions, which are characterized by low precipitation, loose soil structure and frequent strong winds, are especially vulnerable to desertification^2^,^3^. The sparse vegetation there can easily get degraded under extreme weather conditions such as serious drought and storms^1^,^4^, or by improper management regimes like overgrazing, intensive agricultural reclamation and over exploitation of fuel material^4^,^5^. Degraded vegetation may further lead to desertification and trigger sand storms, resulting in serious environmental problems and great economic cost^6^.

China possesses extensive arid and semi-arid areas (3.32 million km^2^), of which 79% located in more than a quarter of the country's territory^7^ (CCICCD, 1997) is highly vulnerable to desertification. The total costs incurred by damage from desertification in China approximate 89.28 billion RMB (around 10.47 billion EUR) annually^4^ and the increase in desertified land is considered a main cause of the drastically increased sand storms in northern China in recent years^6^. To combat desertification and sand storms, the Chinese government has launched large and costly programs (accounting for 0.024% of the country's annual GDP^8^) to fix sand and increase vegetation cover. Among these projects, manual plantation and aerial seeding are commonly applied measures, with the latter being preferred in remote areas. Since 1990, planting and aerial seeding has been applied on 24.7 and 12.7 million hectares, respectively, in China^9^.

In seeding projects, species are selected that can tolerate harsh dune habitats and are able to realize fast population expansion. Clonal plants, which often are successful colonizers in stressful environments^10^,^11^, are commonly used to fix shifting dunes and prevent sand encroachment in adjacent areas in China and many other desertified regions in the world^12^,^13^. While some attention has been paid to the strategies that allow clonal plants to adapt to the harsh dune environment, most work has considered plant performance and reproduction at individual level, without integrating those to the level of populations^10^,^14^,^15^,^16^,^17^. However, assessing the success of the species for re-vegetation requires an integrated approach at population level including the whole life cycle. Demographic models, which integrate all vital rates of all individuals within a population, are an appropriate tool to investigate the life history strategies and population dynamics in varying dune environment. Clonal individuals (ramets) often exhibit large variation in growth and produce offspring of different sizes, due to the heterogeneous dune conditions as well as the genetic differences between clones. This variation is often neglected in plant demographic studies, although it may contribute greatly to population maintenance^18^,^19^. Classical matrix models that are often used for the demographic study of plants offer limited possibilities to include strong variation in growth and offspring size^5^,^18^,^20^. Integral Projection Models (IPMs), an extension of matrix models, allows for explicit inclusion of this variation and are therefore particularly useful to analyze the demography of dune clonal plants^5^,^20^,^21^,^22^,^23^.

Here we used IPMs to study the ramet population dynamics of a rhizomatous clonal shrub *Hedysarum laeve*, which has been widely dispersed by aerial seeding in Mu Us Sandland in northern China since the 1950s^24^,^25^. *Hedysarum laeve* is locally abundant in mobile dunes in the region and plays an important role in trapping sand and protecting dunes from wind erosion^25^. The species shows a high morphological plasticity and clonal integration that enables it to cope with sand burial and heterogeneity of nutrition and water. Yet, little is known about its life history strategy, population growth rate and the regulation of its population growth in the habitat where it was seeded. Filling these knowledge gaps requires a demographic study that considers the entire life cycle^19^,^20^,^26^,^27^. Specifically, we addressed the following questions: 1) What is the rate of growth or decline of natural populations of *H. laeve* in mobile dune environments? 2) Which vital rates are most important for population maintenance? 3) How do *H. laeve* populations respond to temporal fluctuations in climatic conditions?

## Results

### Vital rates in relation to size and age

**Figure 1:**
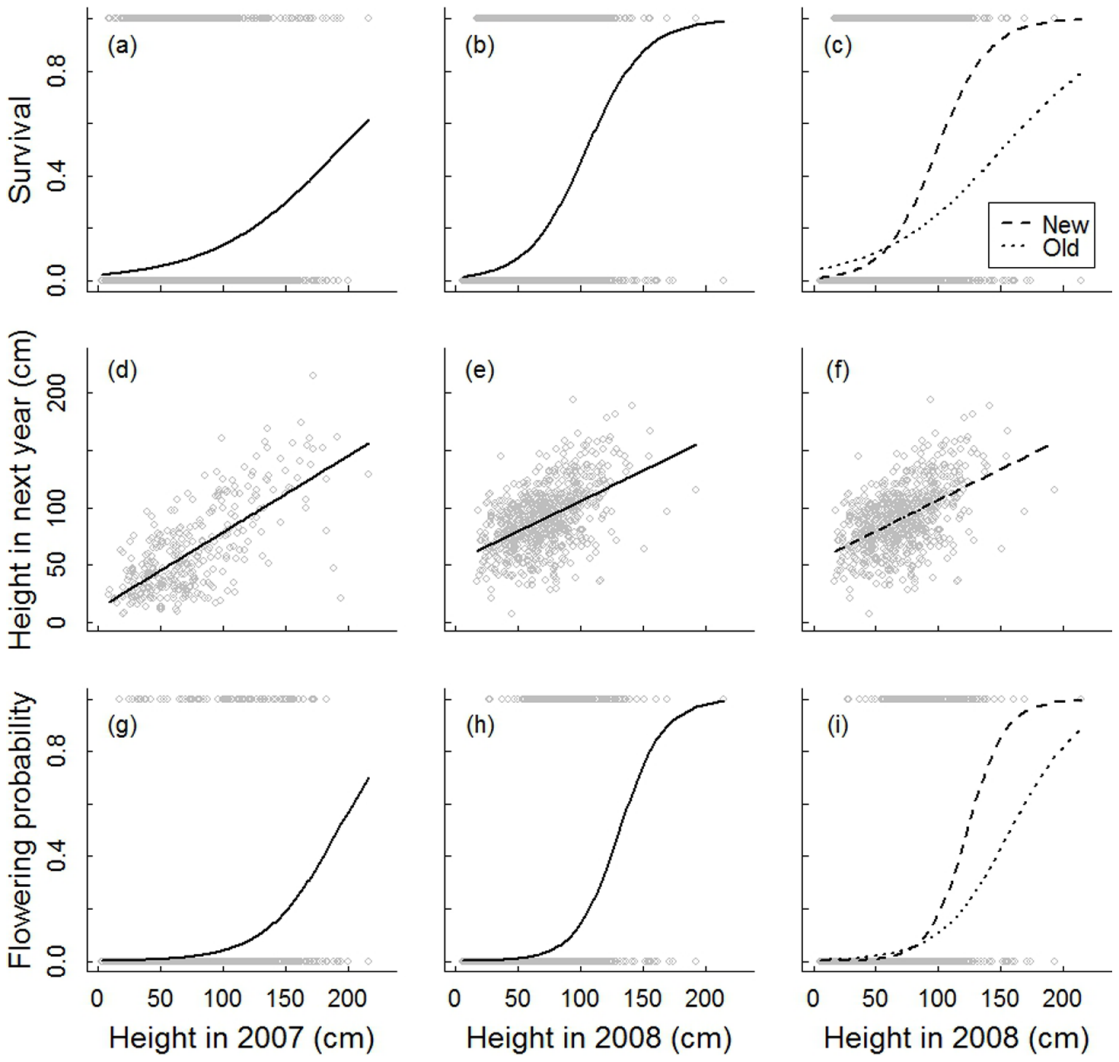
Relations of vital rates with plant height for *Hedysarum laeve* in Mu Us Sandland during 2007–2008 (a, d, g) and 2008–2009 (b, c, e, f, h, i). Individuals in 2008 were presented as one group (b, e, h), but also divided into two groups as new recruited ramets (new) and ramets survived from previous years (old; c, f, i). Regression functions are described in Tables 1 and 2.

Ramet survival of *H. laeve* was generally very low; for example, individuals of 50 cm (the average ramet size) had a survival probability of less than 10% in both census periods (Table 1, Fig. 1a, b). Survival chance increased with ramet size. Large individuals (100–200 cm high) exhibited a considerably higher survival probability, particularly during the second census period (45.1–98.4%; Fig. 1b). Interestingly, newly recruited ramets had a higher survival rate than existing ramets (Table 2, Fig. 1c).

Height growth declined with ramet height, as indicated by the regressions of size in next year vs. size in current year with slopes of <1 (Table 1, Fig. 1d, e). Large growth variation, both positive and negative (shrinkage), was observed during both census periods (Fig. 1d, e). Ramets generally showed slower growth in the first (Fig. 1d) than in the second year (Fig. 1e). There was no effect of age on ramet growth (Table 2, Fig. 1f).

The average proportion of ramets that were flowering was 1.7 and 2.2% in the first and second period, respectively (Table 1). Flowering probability increased with plant height (Fig. 1g, h) and was higher for newly recruited ramets than for old ramets (Table 2, Fig. 1i).

Population recruitment was realized entirely by clonal propagation. Although seeds were produced annually, no seedlings (new or old) were observed during the study period within the permanent plots. Recruitment by seed is possible in the area, as we found two seedlings outside the plots in the first census period, but its contribution to growth of our study populations was negligible. Each ramet produced an average number of 1.4 and 1.0 new ramets in the first and second year, respectively. While fewer ramets were produced during the second year, these recruits were taller than those produced in the first year (Table 1).

### Population growth rates and stable structures

**Figure 2:**
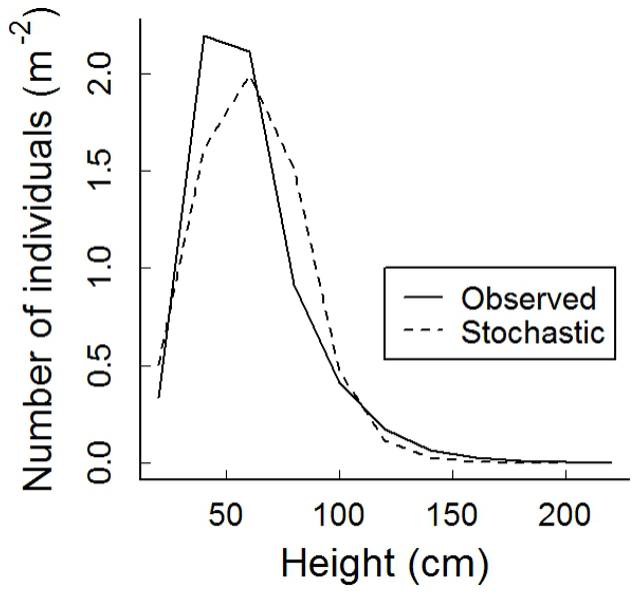
Observed population size structures (Observed) of *Hedysarum laeve* in Mu Us Sandland during 2007–2009, and stochastic size structure (Stochastic) from a stochastic Integral Projection Model.

Projected deterministic population growth rates (λ) were very high in both census periods (2007–2008: λ = 1.27 [1.22, 1.33]; 2008–2009: λ = 1.53 [1.50, 1.60], values in square brackets are confidence intervals). The stochastic population growth rate – that takes the temporal fluctuations in environmental conditions into account – was also high, with a value of 1.32 [1.10, 1.70]. These results suggest that the populations were experiencing fast expansion (i.e., expansion in population size; we did not measure spatial spread). Both the observed and the stochastic population structure were characterized by high proportions of small- and moderate-sized individuals (20–80 cm, Fig. 2). The similarity between the observed size structure and the stable size structure from stochastic simulation was 87%, and the observed structure was more peaked towards smaller sizes (Fig. 2).

### Elasticity analysis and Life Table Response Experiment

**Figure 3:**
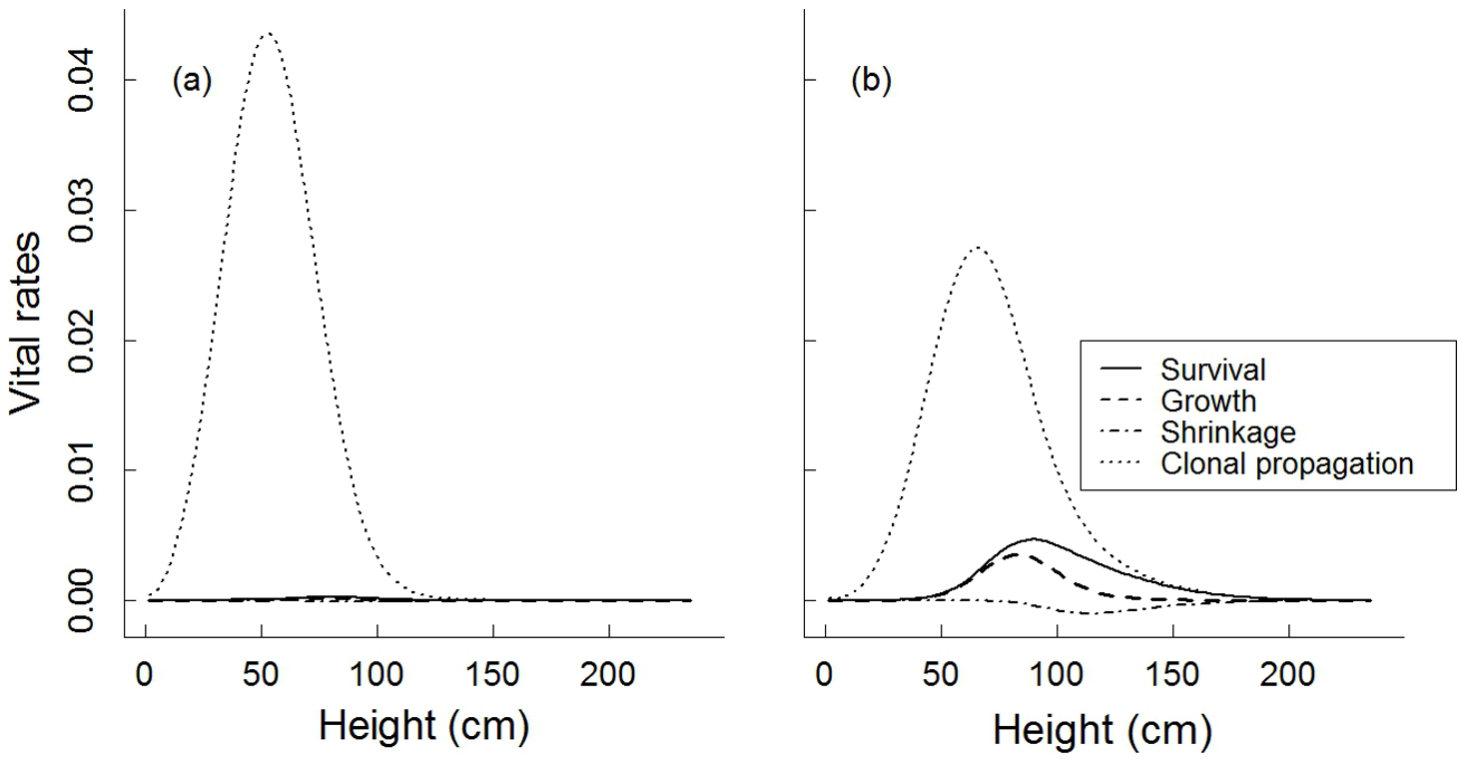
Vital rate elasticity for *Hedysarum laeve* in Mu Us Sandland during 2007–2008 (a) and 2008–2009 (b).

**Figure 4:**
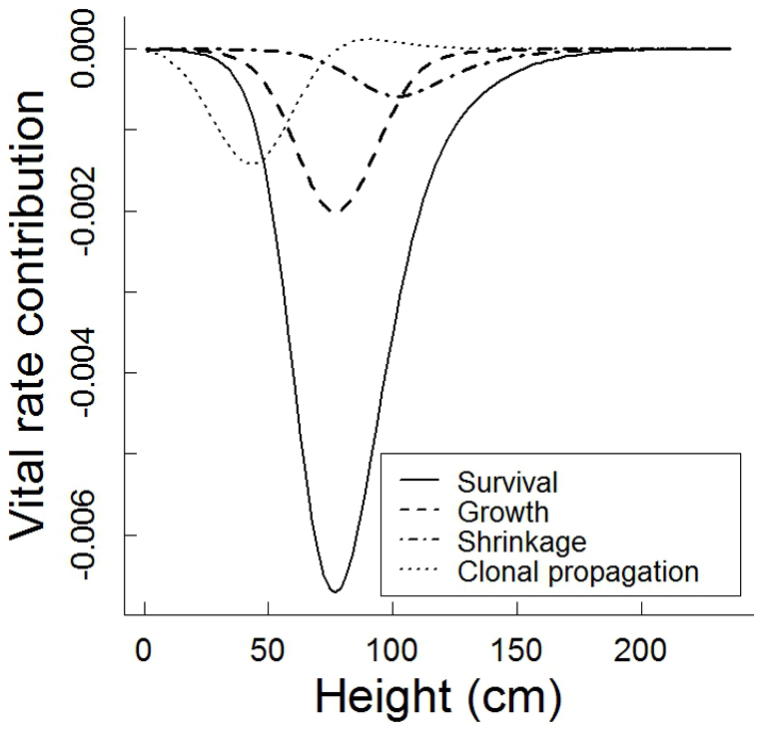
Results of analyses of Life Table Response Experiments (LTRE) for *Hedysarum laeve* in Mu Us Sandland. Shown are the contributions of vital rates to temporal variation in population growth between 2007–2008 and 2008–2009.

Elasticity analyses showed that the population growth rate was most sensitive to changes in clonal propagation (Fig. 3a, b, both years) followed by survival (Fig. 3b, second year, Supplementary Table S1). The analysis of the Life Table Response Experiment (LTRE), which quantifies the contribution of each vital rate to the observed difference in population growth rates, showed that the lower λ in the first census period was attributed mainly to lower survival (explaining 67% of variation), and to a lesser extent to variations arising from growth (17%), shrinkage (10%) and clonal propagation (6%; Fig. 4). The relative contributions of vital rates to variation in population growth rates were distributed unevenly across individuals with different sizes. The contributions of survival and growth were mainly due to plant individuals of 50–100 cm, that of shrinkage mainly due to individuals of 70–150 cm, and that of clonal propagation mainly due to the recruitment of new ramets shorter than 70 cm.

## Discussion

### Reproductive strategies

Although a substantial percentage of *H. laeve* ramets flowered annually, no new seedlings were found within our study plots during the three censuses. The unsuccessful sexual reproduction of *H. laeve* could be caused by failure of part of the flowers to set seeds (S. Li, pers. observ.) or poor seed germination in field. It has been shown that a certain depth of sand burial is required for seed germination of desert plants, especially those with larger seed size^28^. In our study area, the dunes were to some extent fixed with the vegetation coverage of around 30%, and seeds might have failed to germinate due to insufficient sand burial. Rare and irregular seedling recruitment is a common phenomenon in clonal plants^29^,^30^ and has been found in clonal desert species even when seeds are regularly available^3^,^17^.

By mere vegetative reproduction clonal plants can realize successful population regeneration in stressful or highly disturbed environments^17^,^31^,^32^. While the unpredictability of rainfall and frequent sand movement often lead to failed seedling recruitment in arid dune environments, clonal offspring suffers much less from this as it receives support (carbohydrates, water and nutrients) from parent ramets by clonal integration. This clonal integration increases the ability to tolerate resource deficiency and environmental fluctuations^17^,^33^,^34^. While population recruitment of non-clonal plants in arid dunes can be completely blocked in very dry years, clonal plant populations may continue to regenerate vegetatively, making them more resistant to the characteristic strong climatic fluctuations in arid dune environments.

### Explorative strategy

The life history of *H. laeve* was characterized by high ramet turnover, with both high ramet mortality and high ramet recruitment. Our analyses showed that age had a negative effect on ramet survival and flowering probability. However, the ramets of the clonal plant *H. laeve* are not monocarpic, as a small proportion of individuals after flowering are able to survive for many years^35^, therefore the high mortality rate is not due to fatal flowering. The age-induced decline in plant performance has also commonly been found in other clonal plant species^36^,^37^. High turnover of ramets may help to improve genet performance by replacing ageing ramets by young ones, because when ageing ramets die, a substantial part of their resources may be withdrawn and re-allocated to the new, growing ramets^36^. On the other hand, soil nutrients in dune environments are quite poor and usually distributed heterogeneously^14^. Under such conditions, clonal spread via rhizomes assists (long-lived) genets to explore large areas for nutrients and water, while fast ramet turnover avoids local nutrient depletion^14^. Although we have no information on longevity and spatial extent of genets, *H. laeve* likely exhibits the explorative foraging strategy outlined above, which is often adopted by rhizomatous clonal plants in resource-poor conditions^38^,^39^,^40^.

### Population dynamics of *H. laeve* in dune habitats

The populations of *H. laeve* in mobile dunes in our study are expanding very rapidly, with the population size increasing by 27–53% annually. The particularly high population growth rate of *H. laeve* enables this species to quickly colonize mobile dunes and possibly also to realize fast spatial expansion. Elasticity analysis revealed that fecundity (clonal propagation), which remained constantly high regardless of rainfall fluctuations, had the largest contribution to population growth rates in *H. laeve*. This result is different from non-clonal shrub species, for which fecundity is generally found to be of low importance for population growth^5^,^20^,^41^,^42^. However, the different elasticity pattern is not entirely surprising. For non-clonal dune shrubs, sexual reproduction is the only reproductive mode and one that can be greatly affected by rainfall fluctuations; therefore its contribution to population growth can be limited^5^,^20^,^41^. On the other hand, non-clonal dune shrubs are often long-lived, and survival is generally less affected by environmental fluctuations^42^; in this case maintenance of survival over time tends to be more important than production of more offspring, especially in stressful conditions^5^. In the clonal shrub *H. laeve*, vegetative reproduction is also less affected by environmental fluctuations, making constant contributions to population growth. Moreover, the ramets of our study species are rather short-lived as shown by high ramet turnover and the negative effects of age on ramet survival and growth. Thus, the higher dependence of (ramet) population growth on fecundity rather than on survival in *H. laeve* could be a specific demographic feature for ramet dynamics in clonal shrub species to successfully exploit dune environments. Furthermore, analysis of Life Table Response Experiments revealed that variation in population growth between years was mainly caused by variation in survival of individuals of intermediate size (50–100 cm), for which the survival rate in the second year (8.8%–45.1%) was 59.2%–231.1% higher than that in the first year (5.6%–13.6%). However, the mean number of ramets produced by a reproductive individual was only 0.01% lower in the second than in the first year, suggesting that clonal propagation remained rather constant between years and thus had very limited contributions to the observed temporal variations in population growth rates. As suggested by buffering life history theory, less variation in the more important vital rates could promote population growth in a variable environment^43^. Our results suggest that the high, constant clonal propagation is the key demographic component for *H. laeve* to realize fast population expansion in fluctuating arid dune environments.

### Implications for dune fixation management

Increasing vegetation cover to get shifting dunes stabilized is fundamental for combating desertification in inland dune areas^1^,^4^,^13^. Plants selected for re-vegetating bare areas need to effectively trap sand by occurring at high densities and need to quickly colonize new areas by having the capacity to attain high population growth rates. Our study showed that populations of *H. laeve*, once established, were able to realize fast population expansion via high clonal propagation, suggesting that this species is suitable for re-vegetation. However, lack of seedling recruitment, as we found during our three-year monitoring, suggests that initial establishment of *H. laeve* populations at a new site in dune environments could be limited by low seedling establishment. Studies on population dynamics of this species, as presented here, should therefore be accompanied by seeding trials in various dune environments, using different seeding methods. Aerial seeding and manually planting seedlings are two commonly used approaches in *H. laeve* re-vegetation projects^24^,^25^. As establishment of *H. laeve* from germinated seeds seems very limited in our study area, possibly as a result of insufficient sand burial^28^, aerial seeding of *H. laeve* might not be an effective way for ongoing dune fixation when the dune vegetation gets denser. Manual planting of seedlings with early establishment aid, such as watering to avoid seedling mortality in severe drought and artificial barriers to protect young seedlings against strong sand burial and denudation in wind events, can be a more effective way to get new *H. laeve* populations established^25^.

Once established, *H. laeve* seems well adapted to active dunes with sparse vegetation by being able to withstand severe sand burial and wind denudation, which are often detrimental to other dune species that are common at later dune fixation stages^44^,^45^. However, as the vegetation becomes denser and the soil water and nutrient conditions get worse due to increased resource competition^46^, *H. laeve* may gradually lose its prominence within 20–35 years and get outcompeted by other dune species that are better adapted to poor nutrient and water conditions^47^. Under natural conditions dune vegetation previously dominated by *H. laeve* will finally reach a climax stage of deep-rooted shrubs in a matrix of predominantly annual herbs with shallow root systems growing only during the favourable season^24^,^46^. The annual herbs-dominated climax vegetation is very fragile and sensitive to human or natural disturbances. Once disturbed, it will quickly turn to shifting dunes^24^,^46^. Given the facts that the *H. laeve*-dominated vegetation is more resistant to disturbances and environmental fluctuations and that this legume species annually can produce large amounts of forage of high nutritive value and good palatability for livestock, the unmanaged natural succession is not economically and environmentally beneficial^24^,^48^. To keep the *H. laeve* populations from declining, moderate livestock grazing is preferred after *H. laeve* populations get well established. At the same time overgrazing should be prevented as it leads to the decline of *H. laeve* and gets the dunes turn back to shifting sand^5^,^6^,^24^. Our study also showed that changes of vital rates for certain size categories, e.g. changes of survival in intermediate-sized individuals (50–100 cm), could be more influential to population growth rate than changes in other size categories (Fig. 4). Hence, to maintain a constant population growth rate in *H. laeve*, a strong reduction in survival of these intermediate-sized plants should be prevented. As mortality is easy to assess, monitoring the proportion of dead plants of intermediate sizes would be a good indicator of potential variation in population growth rates under different grazing pressures. Since the present study is based on demographic data in three annual censuses, it captures only a part of the environmental variation and population growth rate could be potentially higher or lower during extremely wet or dry years than observed here. Therefore, local weather conditions should also be taken into account when applying our results in practical management.

We found that ramets of *H. laeve* were rather short-lived and annually large number of ramets died before winter. Therefore, the aboveground vegetation of dunes dominated by *H. laeve* can be very sparse in winter and early spring. In order to facilitate the effective fixation of the sand, one may consider adding other dune species, such as *Artemisia ordosica* and *Caragana intermedia* that have large aboveground crown volume, to a *H. laeve*-vegetation^5^,^20^.

## Methods

### Study species and area

*Hedysarum laeve* Maxim. (Fabaceae) is a rhizomatous, clonal shrub with a high capacity of trapping sand. This species is widely distributed in semi-arid sandlands in northern China^24^,^25^. It realizes population recruitment by both sexual and vegetative reproduction^25^. A seedling of *H. laeve* becomes sexually reproductive as from two years after establishment and then also starts vegetative reproduction by sprouting rhizomes. Rhizomes extend horizontally for distances ranging from several centimetres to several metres and then form new ramets^25^.

The study was conducted at Ordos Sandland Ecological Station (OSES, 39°29′37.6″ N, 110°11′29.4″ E) of the Institute of Botany of the Chinese Academy of Sciences, located in the north-eastern Mu Us Sandland in Inner Mongolia, China. Mu Us Sandland is a semi-arid area of 39 800 km^2^, with a mean annual precipitation of 260 to 450 mm, which is mainly concentrated in summer^24^. Our first study period 2007–2008 (hereafter ‘a standard year’) represented average climate conditions at the site, with the rainfall close to the long-term average calculated from the precipitation records available for 1986–2012 (data from KNMI Climate Explorer, http://climexp.knmi.nl). The second study period 2008–2009 was drier (hereafter ‘a dry year’), with the annual rainfall and growing-season rainfall being 13% and 30% lower than the average, respectively. The mean annual temperature is 7.5 to 9.0°C, with a maximum of 20 to 24°C in July and a minimum of −8 to −12°C in January (Zhang 1994).

### Study design and data collection

In 2007, three permanent plots with sizes ranging between 400 m^2^ and 512 m^2^ were constructed in shifting dunes. We chose locations with sparse vegetation (cover around 30%) where *H. laeve* was the main dominant species. The size of the three plots was 1376 m^2^ in total. The plots were subdivided into 334 subplots of 2 × 2 m. For ramets with heights from 20 cm to 60 cm, 262 subplots were sampled. Ramets shorter than 20 cm or taller than 60 cm were searched in the entire plot. A total of 19057 ramets were included (Supplementary Table S1); genets could not be identified in the field because of the long rhizomes between ramets and the large number of ramets that comprise one genet. In the following we will refer to ramets as “individuals”.

Annual censuses were conducted in September 2007, 2008 and 2009. At the first census, total height and basal stem diameter were measured for each individual (ramet), and their reproductive status was recorded. Upon first measurement, each individual was labeled. In 2008 and 2009 the survival of all labelled individuals was checked, and the surviving individuals were re-measured, their reproductive status was recorded and new vegetatively produced ramets within the plots were searched and measured. It proved impossible to trace the ‘parent’ of new ramets without considerable excavation and heavy disturbance of rhizomes and habitat. We therefore assumed that vegetative reproduction was generally size-dependent^49^. Thus, the probability of ramet production from each ramet individual was calculated by multiplying its size (height) by the ratio of the total number of newly recruited ramets in the current year to the sum of sizes of all ramets in the previous year in each plot. Seedling recruitment was monitored during the entire growing season from May to September in each census.

### Statistical analyses

We used regression models to relate current size (*x*) of an individual to its future size, survival and reproduction. The future plant size (*μ*(*x*)) and variance of growth (*σ*^2^ (*x*)) were fitted with linear regressions, and the probability of survival *s*(*x*) and of flowering *p_f_*(*x*) were fitted by logistic regressions, with plant size as explanatory variables. As regression models with height as size variable yielded higher *R*^2^ values than models based on stem diameter, we used height to characterize plant size in all models. Moreover, we tested the effects of age on individual survival, growth and flowering probability with multiple regressions. Individuals recorded in 2008 were classified into one of two groups: those newly recruited in 2008 and those that survived from years previous to 2008. As we were unable to assign age to individuals in 2007, we lacked information to calculate transitions among age classes and did not include age in our population models.

### Integral Projection Models

We analyzed the population dynamics of *H. laeve* using Integral Projection Models (IPMs), which describe how a continuously size-structured population changes in discrete time^21^. In IPMs, the state of the population at time *t* is described by a distribution function *n*(*x*, *t*) and *n*(*x*, *t*)*dx* represents the number of individuals with size in the range [*x*, *x* + *dx*]. The population dynamics is then written as: 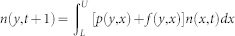
where [L, U] is the range of all possible sizes, *p*(*y*, *x*) represents survival and growth from size *x* to size *y*, and reproduction *f*(*y*, *x*) represents the number of ramet recruits of size *y* at *t* + 1 produced by parent ramets of size *x*. *p*(*y*, x) was calculated as *p*(*y*, *x*) = *s*(*x*) *g*(*y, x*), where *s*(*x*) is survival and *g*(*y*, *x*) is a normal probability density function with mean future plant size *μ*(*x*) and growth variance *σ*^2^ (*x*). *f*(*y*, *x*) was calculated as *f*(*y*, *x*) = *f_n_*(*x*) *f_d_*(*y*), where *f_n_*(*x*) is the number of new ramets produced by a parent ramet at size (*x*) and *f_d_*(*y*) is the size distribution of new ramets. *p*(*y*, *x*) + *f* (*y*, *x*) is called the kernel, *k*(*y*, *x*), a non-negative surface representing all possible transitions from size *x* to size *y*.

The kernel *k*(*y, x*) can be transformed into a large transition matrix **K**(*y*, *x*) with *w* categories, using the midpoint rule^21^. The dynamics of the population can then be described as in a classical matrix model: **n**(*t* + 1) = **K** **n**(*t*), that can yield the same output as matrix models: population growth rate (λ), sensitivity and elasticity^21^. We used 100 mesh points, because λ values in all populations hardly changed any more when further increasing the number of mesh points. Because we found significant differences between periods, we constructed an IPM for each census period separately.

Confidence intervals of λ were calculated by bootstrapping^50^. The same number of individuals as recorded (19057) was resampled with replacement from the data matrix, and all regression coefficients for relationships between vital rates and plant size and habitat were recalculated and used to obtain λ for each of 5000 bootstrap replicates.

Stochastic population growth rates (λ_s_) were calculated by selecting either a dry-year or standard-year kernel with the probability of 26% and 74% for each of 10000 model iterations. These probabilities were chosen based on long-term climate records during 1986–2012, in which the growing-season rainfall was equal to or lower than that in our dry year in seven out of 27 years (data from KNMI Climate Explorer, http://climexp.knmi.nl/get_index.cgi). We then calculated the geometric means of the obtained annual growth rates after excluding the first 200 transient iterations^51^, and obtained 95% confidence intervals for λ_s_ from its frequency distribution.

To examine whether the observed plant size distribution deviated from the expected one, we compared the observed population structures (mean of the three annual censuses) to the stable structures resulting from stochastic IPMs, using the percentage similarity index PS^52^: PS = Σ(min[obs_i_, ssd_i_]) × 100, where obs_i_ and ssd_i_ are vectors of observed population structures and stable size distributions, respectively (both vectors scaled to sum to 1). High values of this index indicate a high level of similarity^53^.

To examine the relative importance of each vital rate to λ, we conducted an elasticity analysis, in which we separated survival from growth and shrinkage^54^. Elasticity quantifies the impact of a proportional change in a vital rate on the proportional change of λ and can be used to guide management^55^,^56^. Differences in population growth rates between periods may be caused by temporal variation in vital rates. Analysis of Life Table Response Experiments (LTRE) allows quantification of the contribution of each element or vital rate to the observed difference in population growth rate^56^. We conducted a one-way design LTRE on vital rates to evaluate the contribution of variation in vital rates to temporal differences in population growth rate^56^.

All analyses were performed with the software R 3.0.1^57^.

## Author Contributions

S.L.L., F.H.Y., M.J.A.W., M.D. and P.A.Z. conceived and designed the investigation. S.L.L. conducted the field work, performed data analyses and wrote the manuscript. F.H.Y., M.J.A.W., M.D., H.J.D. and P.A.Z. commented on the manuscript and contributed to revisions.

## Supplementary Material

Supplementary Information Supplementary Table S1

## Figures and Tables

**Table 1:** Statistical models and parameter estimates used to construct the kernel for the Integral Projection Models of *Hedysarum laeve* in Mu Us Sandland during 2007–2008 (period 1) and 2008–2009 (period 2). The models are functions of plant height (*x*, cm). Values in parentheses are standard errors of the parameter estimates. *p* < 0.01 indicates the slope and intercept in the regression models are significantly different from zero

Demographic process	Period	Model
Survival probability (*s*)	1	Logit(*s*) = −3.83 (0.12) + 0.02 (0.00)*x*n = 4989, R^2^ = 0.09, *p* < 0.0001
	2	Logit(*s*) = −4.49 (0.11) + 0.04 (0.00)*x*n = 7214, R^2^ = 0.21, *p* < 0.0001
Flowering probability (*p*_f_)	1	Logit(*p*_f_) = −6. 62 (0.28) + 0.03 (0.00)*x*,n = 4989, R^2^ = 0.24, *p* < 0.0001
	2	Logit(*p*_f_) = −7. 50 (0.27) + 0.06 (0.00)*x*,n = 7214, R^2^ = 0.32, *p* < 0.0001
Future size (*μ*)	1	*μ* = 11.72 (3.14) + 0.67 (0.04)*x*,n = 336, R^2^ = 0.50, *p* < 0.0001
	2	*μ* = 52.76 (2.49) + 0.53 (0.03)*x*,n = 745, R^2^ = 0.25, *p* < 0.0001
Variance of growth (*σ*^2^)	1	*σ*^2^ = 11.2 (1.64)*x*,n = 336, R^2^ = 0.12, *p* < 0.0001
	2	*σ*^2^ = 6.72 (1.21)*x*,n = 745, R^2^ = 0.04, *p* < 0.0001
Size distribution of new ramets	1	Gaussian with mean = 44.6, Variance = 467.8, n = 6878
	2	Gaussian with mean = 56.9, Variance = 480.1, n = 7179

**Table 2:** Effects of age on survival, growth and flowering probability of *Hedysarum laeve* in Mu Us Sandland during 2008–2009. The models are functions of plant height (*x*, cm), age (a) and their interaction (*x*_a). Values in parentheses are standard errors of the parameter estimates. *p* < 0.01 indicates the slope and intercept in the regression model are significantly different from zero

Demographic process	Model
Survival probability (*s*)	Logit(*s*) = −4.77 (0.12) +1.60 (0.37)a + 0.05 (0.00)*x* − 0.03 (0.00)*x*_an = 7214, R^2^ = 0.22, *p* < 0.0001
Flowering probability (*p*_f_)	Logit(*p*_f_) = −8.10 (0.32) + 2.36 (0.78)a + 0.07 (0.00)*x* − 0.03 (0.01)*x*_a,n = 7214, R^2^ = 0.33, *p* < 0.0001
Future sizes (*μ*)	*μ* = 52.21 (2.62) + 0.54 (0.04)*x*,n = 745, R^2^ = 0.25, *p* < 0.001
