# Supplementary material for: Mobile dune fixation by a fast-growing clonal plant: a full life-cycle analysis
Source: Sci Rep. 2015 Mar 11;5:8935. doi: 10.1038/srep08935 (PMC4355633; doi:10.1038/srep08935)
Supplement: Supplementary Information — Supplementary Table S1 [file srep08935-s1.doc]

**Supplementary Information**

**Mobile dune fixation by a fast-growing clonal plant: a full life-cycle analysis**

Shou-Li Li^*^, Fei-Hai Yu, Marinus J.A. Werger, Ming Dong^*^, Heinjo J. During and Pieter A. Zuidema

*Corresponding authors

<mailto:> [shouli.s.li@gmail.com](mailto:shouli.s.li@gmail.com) (S.-L. Li), dongming@ibcas.ac.cn (M. Dong)

**Supplementary Table S1.** Summary of dataset and parameter estimates based on Integral Projection Models for *Hedysarum laeve* in Mu Us Sandland during 2007-2008 and 2008-2009

| Measure | 2007-2008 | 2008-2009 |
| --- | --- | --- |
| Number of individuals recorded | 11868 | 14393 |
| Number of individuals died | 4653 | 6469 |
| Number of individuals survived | 336 | 745 |
| Number of newly recruited individuals | 6878 | 7179 |
| Mean height of new individuals (cm) | 44.6 | 56.9 |
| Population growth rates (λ) | 1.27 [1.22, 1.33] | 1.53 [1.50, 1.60] |
| Elasticity of survival | 0.006 | 0.130 |
| Elasticity of positive growth | 0.003 | 0.066 |
| Elasticity of negative growth | -0.002 | -0.024 |
| Elasticity of clonal propagation | 0.913 | 0.685 |
